# Association between copy number alterations estimated using low-pass whole genome sequencing of formalin-fixed paraffin-embedded prostate tumor tissue and cancer-specific clinical parameters

**DOI:** 10.1038/s41598-023-49811-w

**Published:** 2023-12-17

**Authors:** Paul Vinu Salachan, Benedicte Parm Ulhøi, Michael Borre, Karina Dalsgaard Sørensen

**Affiliations:** 1 Department of Molecular Medicine, Aarhus University Hospital, Aarhus N, Denmark; 2 Department of Clinical Medicine, Aarhus University, Aarhus N, Denmark; 3 Department of Pathology, Aarhus University Hospital, Aarhus N, Denmark; 4 Department of Urology, Aarhus University Hospital, Aarhus N, Denmark

**Keywords:** Prostate cancer, Cancer genomics

## Abstract

Copy number alterations (CNAs) are frequently observed in early-stage prostate cancer and are associated with disease recurrence and tumor aggressiveness. Cost-effective assessment of CNAs could enhance clinical utility of CNAs. Here, we combined the cost-effectiveness of low-pass (low coverage) whole genome sequencing (LPWGS) and the routine availability of formalin-fixed paraffin-embedded (FFPE) tumor tissue for assessing CNAs in a cohort of 187 men with early-stage localised prostate cancer. We detected well known CNAs in 8p, 8q, 13q and 16q and recurrent gains of the oncogene *MYC* and losses of the tumor suppressor genes *NKX3-1*, *PTEN* and *RB1*, indicating assay reliability. The estimated burden of CNAs was significantly associated with Gleason score, pathological T stage, surgical margin status and biochemical recurrence. Further, genomic losses or gains in specific chromosomal arms were significantly associated with worse BCR-free survival. Copy number signatures extracted from the LPWGS data showed potential for risk stratifying patients, where signatures S1 and S2 showed significant association to worse BCR-free survival compared to S3. Our study provides clinical validation of the associations between CNAs and tumor aggressiveness in an independent and representative RP cohort, while demonstrating the feasibility of performing LPWGS of FFPE tumor tissue for cost-effective assessment of CNAs.

## Introduction

With an estimated 1.4 million new cases worldwide in 2020, prostate cancer (PCa) remains the second most commonly diagnosed non-skin cancer among men^1^. Localized (i.e. early stage) PCa is curable by radical prostatectomy (RP), but about 30% of the patients experience disease relapse (biochemical recurrence; BCR) within a 5 to 10-year period^2,3^ as indicated by a rise in serum prostate specific antigen (PSA) levels. Current prognostic tools at initial diagnosis of PCa (e.g. PSA, Gleason) are sub-optimal for risk stratification and cannot clearly distinguish aggressive from indolent PCa cases, or are cost-prohibitive in the routine clinical setting (e.g. whole genome (WGS) and whole exome (WES) sequencing). This necessitates the need for better and cost-effective prognostication strategies for risk stratifying men with localized PCa.

Even at the early stages, many prostate tumors exhibit perceivable genomic instability in the form of copy number alterations (CNA)^4,5^. Genomic losses of chromosome arms 8p and 13q, and gain of arm 8q are among the most frequent alterations seen in PCa^6–9^. The percentage of the genome altered (PGA) reflects the CNA burden across the entire tumor genome^10^ and can be used as a measure of genome instability. Studies have reported the clinical potential of using CNA burden as a biomarker for predicting BCR and overall survival (OS) among early stage PCa patients^10^. Recently, copy number signatures were reported to predict progression-free (PFS) and overall (OS) survival in men with early-stage and advanced (metastatic) PCa^11^ using published deep WES data.

In this study, we combined the cost-effectiveness of low-pass WGS with the routine availability of FFPE prostate tumor tissue to profile CNA in men with localized PCa. We analysed the association between genome instability (as assessed from low coverage sequencing data) and routine clinical parameters know to be associated with PCa aggressiveness in a systematic manner using a cohort of 187 patients with localized PCa. We demonstrate the feasibility of using LPWGS on FFPE prostate tumor tissue samples for copy number assessment. We re-identified known recurrent alterations in early stage PCa and provide clinical validation of the associations between CNA burden and tumor aggressiveness in a representative and independent RP cohort using a novel methodology (LPWGS).

## Results

### LPWGS of FFPE tumor tissue for assessing copy number alterations

We used PCa tissue samples obtained at RP from 187 patients with clinically localized disease for copy number profiling. The median age at RP for the patients was 64.2 years and the median PSA was 12.1 ng/ml (Table 1). About two-thirds of the patients (120/187, 64.2%) had a Gleason score greater than or equal to 7 and about one-third (67/187, 35.8%) had a pathological T-stage greater than or equal to T3a (Table 1). Complete clinicopathological characteristics for the cohort is given in Table 1.

**Table 1:** Clinicopathological characteristics for the cohort.

Characteristic	
Patients, *N*	187
Age at RP (years)	
Median (range)	64.2 (48.3–76.8)
PSA at diagnosis (ng/ml)	
Median (range)	12.1 (2–61)
Pathological Gleason score	
< 7, *N* (%)	67 (35.8)
= 7, *N* (%)	88 (47.1)
> 7, *N* (%)	32 (17.1)
Pathological T-stage	
T2a, *N* (%)	23 (12.3)
T2b, *N* (%)	17 (9.1)
T2c, *N* (%)	80 (42.8)
T3a, *N* (%)	46 (24.6)
T3b, *N* (%)	21 (11.2)
Surgical margin status	
0, *N* (%)	125 (66.9)
1, *N* (%)	62 (33.1)
Biochemical recurrence status	
BCR, *N* (%)	96 (51.3)
BCR-free, *N* (%)	91 (48.7)
Total follow-up (months)	
Median (range)	134.2 (12.1–249.3)

Sequencing reads were aligned to the human reference genome (hg38). We obtained a median coverage of 0.1X (range: 0.02X-0.48X) across the entire cohort (Fig. S1A). We estimated genome-wide copy number across 50 kb bins using CNAclinic^12^. Low and similar MAPD scores (median = 0.19, median absolute deviation = 0.02) were obtained for the majority of the samples (Fig. S1B), indicating data quality and consistency across the cohort.

**Figure 1:**
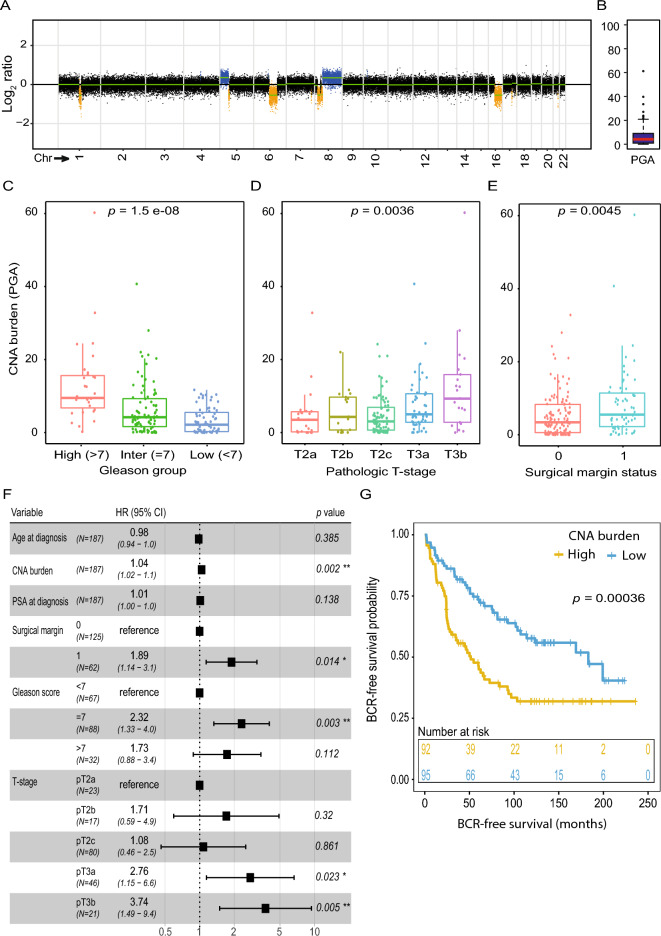
(**A**) Genome-wide copy number plot for a representative patient with a sequencing coverage of 0.1 × showing known PCa-specific genome alterations. (**B**) Boxplot showing distribution of CNA burden in this early stage PCa cohort. (**C**) Boxplots showing association between CNA burden and Gleason score. *P*-value calculated from Kruskal–Wallis test. (**D**) Boxplots showing association between CNA burden and pathologic T-stage. *P*-value calculated from Kruskal–Wallis test. (**E**) Boxplots showing association between CNA burden and surgical margin status. *P*-value calculated from Wilcoxon test. (**F**) Forest plot showing multivariate analysis of association between CNA burden and BCR, controlling for age, PSA, Gleason score, and pathologic T-stage. *P*-value calculated from Wald test. (**G**) Kaplan–Meier plot of association between dichotomized CNA burden and BCR-free survival. *P*-value calculated from log-rank test. *Chr* chromosome, *PGA* percent genome altered, *Inter* intermediate, *HR* hazard ratio.

Known PCa-associated genomic alterations were identifiable in most copy number altered samples (e.g. Fig. 1A), including recurrent gains in chromosome arm 8q^5^, harboring the oncogene *MYC*, and losses in chromosome arms 8p, 10q, and 13q^5,9^, harboring known tumor suppressor genes NK3 homeobox 1 (*NKX3.1*), Phosphatase and tensin homolog (*PTEN*), and Retinoblastoma 1 (*RB1*), respectively. This indicated that LPWGS of FFPE tumor tissue could provide sufficient resolution to estimate recurrent CNA events.

To further generalize the copy number calls, we calculated the CNA burden (percent genome altered) for each patient (Fig. S1C) as a measure of genome instability. The median CNA burden for our cohort was 4.2% (range 0.0–60.2%; Fig. 1B), which is similar to the median estimates (0.7–6.2%) from prior studies of localized PCa^10,13^, and to the CNA burden estimated from SNP array analysis in The Cancer Genome Atlas Prostate Adenocarcinoma (TCGA-PRAD) dataset (median = 4.6%) and by WES analysis in the TCGA-PRAD dataset (median = 3.2%). No significant correlation was observed between CNA burden estimated using LPWGS in our study and the sequencing coverage (Fig. S1D) or MAPD scores (Fig. S1E). Further, when comparing to the clinical and pathological characteristics, high CNA burden in our cohort was significantly associated with higher Gleason score (*p* = 1.5e−08; Fig. 1C), higher pathological T stage (*p* = 0.0036; Fig. 1D), and a positive surgical margin (*p* = 0.0045; Fig. 1E), corroborating the correlation between high CNA burden and an aggressive disease.

In addition, a higher CNA burden was significantly associated with BCR (HR 1.06; *p* = 2.9e−07) in univariate cox regression analyses. After controlling for routine clinicopathological variables (age, PSA, Gleason score, T stage, and surgical margin status), CNA burden remained a significant independent predictor of BCR (HR 1.04; *p* = 0.002; Fig. 1F). Finally, when dichotomised based on the median CNA burden, patients with a high CNA burden showed worse BCR-free survival compared to patients with low CNA burden (*p* = 0.00036; Fig. 1G) in Kaplan–Meier analysis.

Taken together, our results indicate this as a representative RP cohort of localized PCa and confirm the prognostic potential of CNA burden estimated from low coverage WGS of FFPE tumor tissue.

### Recurrent alterations identified from LPWGS of FFPE tumor tissue associate with BCR

**Figure 2:**
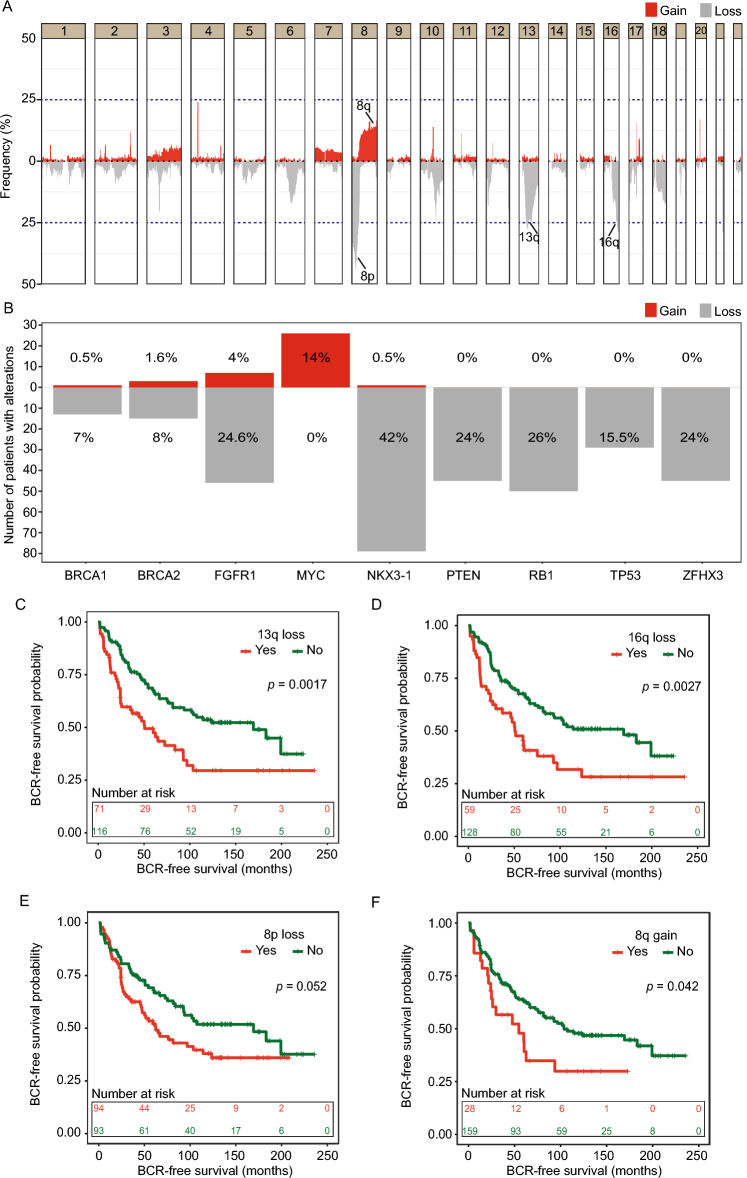
(**A**) Cohort-wide CNA frequency plotted for each 50 kb genomic bins. Well known PCa-specific recurrent alterations are marked. (**B**) Number of patients in our RP cohort with alterations in genes that were previously reported to be recurrently affected by CNA in early stage PCa and located in regions 8p, 8q, 13q, and 16q. The percent tumors with gains or losses observed across the entire cohort are shown for each gene against the bars. (**C**–**F**) Survival plots showing BCR-free survival probability for patients dichotomised based on the presence (Yes) or absence (No) of losses within 13q (**C**), 16q (**D**), and 8p (**E**), and gains within 8q (**F**). *P*-values are calculated from log-rank test.

Next, we plotted CNA frequencies for each 50 kb bin genome-wide for the entire cohort (n = 187; Fig. 2A). We identified recurrent losses in 8p, 13q, and 16q^5,7,9^, and gains in 8q^5,9^ among the most frequent CNAs in our cohort, confirming this as a representative RP cohort. These regions harbour well known PCa-associated tumor suppressor genes (*NKX3.1*, *RB1*) and oncogenes (*MYC*). In agreement, assessment of specific genes showed that alterations in these tumor suppressor genes and oncogenes occurred in high frequencies in our cohort with loss of *NKX3.1* observed in 79 (42%) samples, loss of *RB1* observed in 50 (26%) samples and *MYC* amplifications observed in 26 (14%) samples (Fig. 2B). Analysis of the TCGA-PRAD Affymetrix SNP array copy number data from 497 early-stage prostate cancer patients also revealed similar cohort-wide alteration frequencies in these genes (Fig. S2A), supporting the results from our LPWGS data. However, compared to both our LPWGS copy number data and the TCGA-PRAD SNP array copy number data, the copy number profiles from the TCGA-PRAD WES data (n = 498) from the same cohort showed higher proportions of amplifications in these genes within the cohort (Fig. S2B), indicating the WES data might be calling more false positive amplifications and suggesting LPWGS as a better alternative to WES.

We next, stratified the patients based on the presence or absence of genomic losses within 8p, 13q, or 16q, respectively, as these were the most frequently detected copy number losses in our cohort (Fig. 2A) and in the literature^6–9^. Survival analyses with this stratification showed that losses in these arms were significantly associated with worse BCR-free survival for both 13q (*p* = 0.0017; Fig. 2B) and 16q losses (*p* = 0.0027; Fig. 2C), whereas a non-significant (*p* = 0.052) trend towards worse BCR-free survival was observed for patients with 8p loss (Fig. 2D). Copy number losses in these chromosome arms have also been reported to associate with poor prognosis in early stage PCa from previous studies using fluorescence in-situ hybridization (FISH)^6,7,14,15^. However, since these studies have relied only on the hybridization to few genes per chromosome arm, this technique is inferior to the resolution achieved by LPWGS. Thus, our study expands on these previous studies and provides validation of these earlier findings in an independent and representative RP cohort using a novel and cost-effective methodology.

While none of the 50 kb genomic bins showed gains above a frequency of 25% in our cohort, recurrent gains in 8q have been described in the literature and previously associated with poor prognosis in early-stage PCa^5,6^. Stratifying the patients in our cohort with or without gains restricted within 8q, we saw a significant (*p* = 0.042) association between 8q gains and worse BCR-free survival in early stage PCa patients in our cohort (Fig. 2E). Taken together, these results indicate the general usability of LPWGS of FFPE prostate tumor tissue in calling recurrent and clinically relevant genomic alterations.

### Additional copy number features identified by LPWGS associate with adverse disease characteristics

Recent studies have interrogated the potential for extracting additional features from genome-wide copy number profiles, as a novel means to develop better prognostic biomarkers in several cancer types^16,17^, including PCa^11^. While one earlier PCa study^11^ used deep WES data for feature extraction, no prior studies have tested this approach for LPWGS data in PCa.

**Figure 3:**
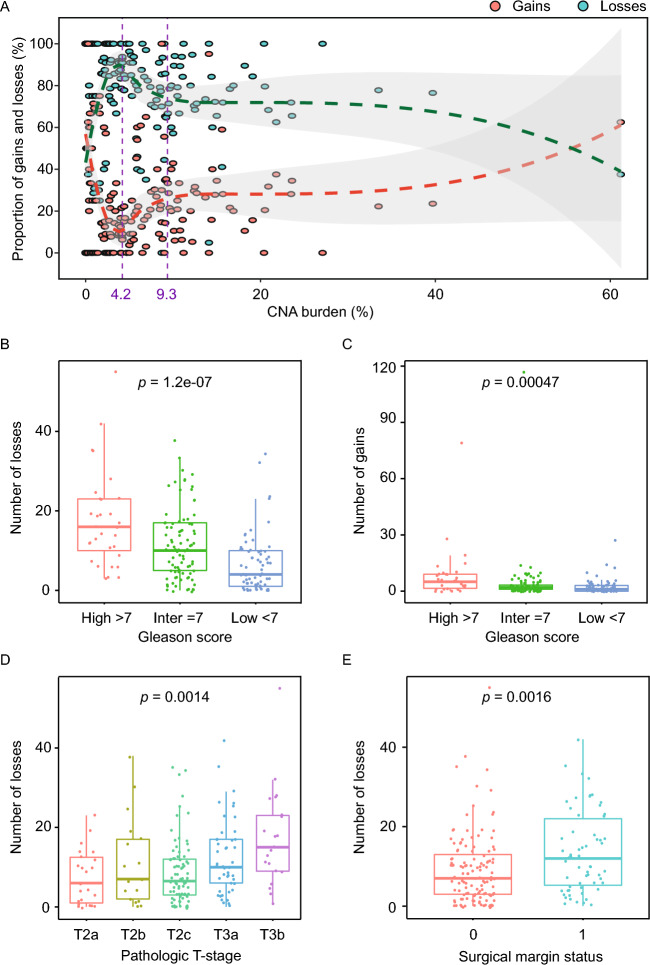
(**A**) Proportion of copy number gains and losses across all patients with CNA (n = 180) ranked according to their CNA burden. Curved dashed lines represent the smoothed conditional means calculated using the *loess* method. Vertical dashed lines represent the median (4.2%) and third quartile (9.35%) of the CNA burden. (**B**–**E**) Boxplots showing association between number of genomic losses and Gleason score (**B**), number of genomic gains and Gleason score (**C**), number of genomic losses and pathological T-stage (**D**), and number of genomic losses and surgical margin status (**E**). *P*-values are calculated from Kruskal–Wallis test (**B**–**D**) or Wilcoxon test (**E**). *Inter*, intermediate.

Thus, we extended this approach to our representative cohort of RP patients for whom we had performed LPWGS. Using sigminer^11^, we first summarised the copy number profiles in terms of basic features such as total number of copy number losses and gains. Contribution of copy number losses to the CNA burden seemed to increase exponentially until the median CNA burden (4.2%), after which a gradual increase in gains was observed at higher CNA burdens and reached stability beyond the third quartile of CNA burden (9.35%, Fig. 3A), suggesting prevalence of early losses. When assessing the association with pathological parameters, for both losses and gains, a higher number of either feature was significantly associated with higher Gleason scores (losses: *p* = 1.2e−07; Fig. 3B, and gains: *p* = 0.00047; Fig. 3C). Further, a higher number of losses was significantly associated with higher pathological T stages (*p* = 0.0014; Fig. 3D) and positive surgical margin (*p* = 0.0016; Fig. 3E), whereas no such association was observed for copy number gains (*p* < 0.05; Fig. S2C,D), indicating that genomic copy number losses are a more prominent adverse feature of PCa. Our results thus expand on previous reports^4^ of association of copy number losses with adverse clinicopathological features and provide independent validation in a representative RP cohort and using a novel methodology.

**Figure 4:**
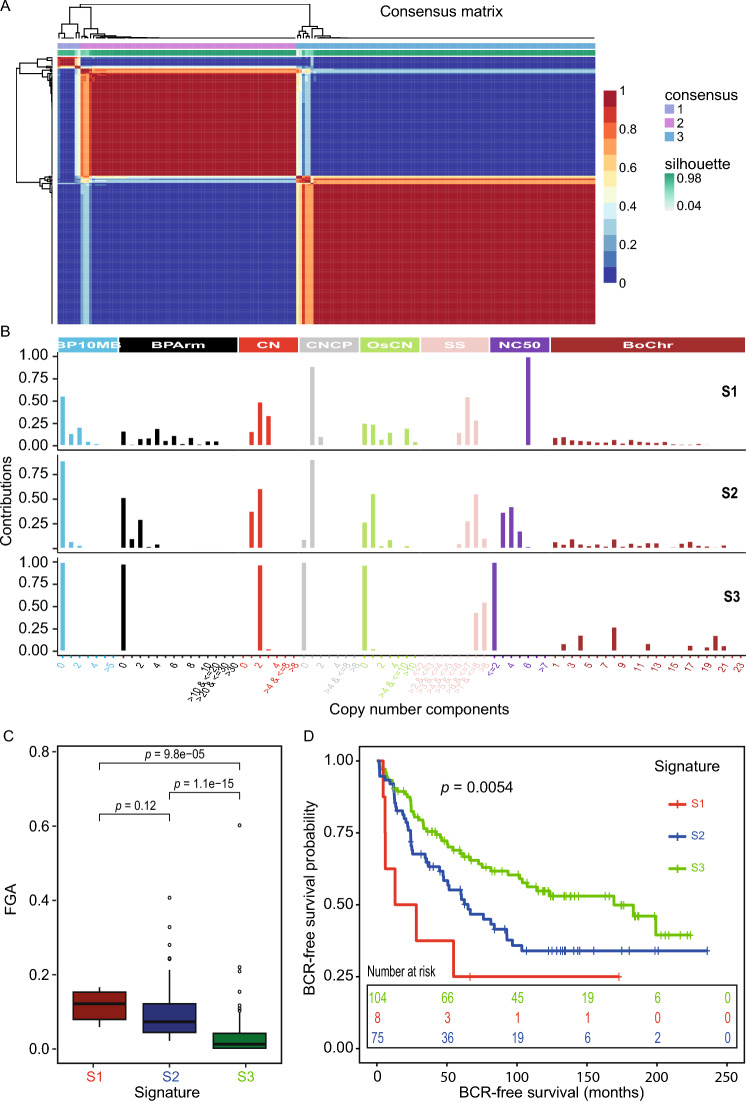
(**A**) NMF clustering of patients based on copy number feature components. 3 distinct clusters were identified. (**B**) Contribution of different copy number feature components to the three signatures S1 (top), S2 (middle), and S3 (bottom). (**C**) Boxplot showing differences in genome instability as estimated using FGA between copy number signature groups. *P*-value from Wilcox test. (**D**) Survival plot showing differences in patient BCR-free survival based on copy number signature. *P*-value from log-rank test.

Next, using sigminer^11^, we extracted eight biologically relevant copy number features and classified them into 80 copy number components, based on the distributions of each feature (see materials and methods) as described in the original study^11^. For example, the number of breakpoints per 10 Mb (BP10 Mb) was classified into six components: 1, 2, 3, 4, 5 and > 5 breakpoints. For each tumor, the value for each component was counted and a tumor by component matrix was created across the entire cohort. The tumor by component matrix was subjected to non-negative matrix factorization and clustering. Based on the cophenetic and RSS scores (Fig. S2E), three clusters were selected as the optimum (Fig. 4A). Signatures underlying these clusters, comprising distinct component patterns were further extracted, resulting in signatures S1, S2, and S3 (Fig. 4B). The most discriminatory feature within the signatures was the copy number (i.e. feature CN; Fig. 4B). S1 captured both copy number gains and losses (Fig. 4B; top) and was observed in 8 patients, whereas S2 captured mainly copy number losses (Fig. 4B; middle) and was observed in 75 patients, suggesting these two signatures might be capturing aggressive disease traits associated with higher genomic instability (i.e. higher FGA; Fig. 4C). S3 appeared mostly copy number neutral (Fig. 4B; bottom) and was observed in the majority of the patients (n = 104), indicating this as a favourable signature characterized by lower levels of genomic instability (i.e. lower FGA; Fig. 4C). Moreover, S1 and S2 had significantly higher FGA when compared to S3 (Fig. 4C) confirming the aggressiveness of these tumor subtypes. In agreement, survival analysis with BCR as a clinical endpoint, showed that patients with S1 and S2 signatures had significantly (*p* = 0.0054) worse BCR-free survival as compared to patients with an S3 signature (Fig. 4D). While S1 showed a trend towards worse prognosis when compared to S2, this difference was not statistically significant in cox regression analysis (*p* = 0.14).

Notably, both S1 and S2 subtypes had more breakpoints per 10 Mb genomic region (BP10MB) and per chromosome arm (BParm), and had shorter segment sizes (SS) as compared to S3 (Fig. 4B). S1 showed the highest value for NC50, followed by S2, and the least in S3 (Fig. 4B). Alterations in chromosomes 4, 8 and 20 contributed more to S3, whereas genome-wide alterations were present in S1 and S2 (Fig. 4B). Taken together, these results indicate the potential for complex signature extraction using LPWGS of FFPE tumor tissue, and provides proof of principle that such complex signatures may be used to identify clinically relevant subtypes of early stage PCa associated with different prognosis.

## Discussions

Clinical utility of a biomarker is often restricted by assay cost. Deep WGS and deep WES provides accurate estimation of genomic copy number alterations in tumor tissue samples^16^, but are generally cost-prohibitive in large-scale clinical settings. Low-cost LPWGS has been indicated as a suitable alternative to deep WGS for profiling CNA^18^. The potential for LPWGS of FFPE DNA for copy number profiling of prostate tumor tissue is still being explored^19^. Large scale pan cancer studies of CNAs have mostly relied on deep WGS, deep WES and array-based data^16,20^. To our knowledge, no study has used LPWGS with a coverage of 0.1X (median coverage in this study) for copy number profiling of FFPE tumor tissue in early stage PCa. We were able to detect aneuploidy in chromosome arms known to be recurrently affected by CNA in PCa^5,9^. These included e.g. the losses in chromosome arms 8p, 13q, and 16q, among the most frequent alterations in our cohort, speaking to the usability of LPWGS of FFPE prostate tumor tissue for clinical research.

We were also able to provide clinical validation of the expected associations between genome instability and adverse clinicopathological characteristics using a representative and independent RP cohort and a novel methodology (LPWGS). Specifically, we show that high CNA burden was associated with high Gleason score, higher tumor stages, positive surgical margin status, and a higher risk of disease recurrence (BCR). CNA burden has previously been reported to be a pan-cancer prognostic factor (including in PCa) that is associated with disease recurrence and death^13^. Interestingly, in our cohort, these associations seemed to be driven by genomic losses more so than by genomic gains, where we also observed an exponential increase in the number of genomic losses contributing to the first and second quartile CNA burdens, after which a gradual decrease and subsequent levelling-off in contributions between genomic losses and gains was observed. In a recent study^19^, genomic losses were also reported to be the major contributor to the first and second quartile CNA burden in men with high risk node negative (M0N0; defined as having at least two of tumour stage category T3/4, prostate-specific antigen (PSA) ≥ 40 ng/ml, or Gleason sum score 8–10) or node positive (M0N1) and metastatic (M1) PCa recruited to the control arm of the STAMPEDE trial (NCT00268476). In contrast, our cohort was more representative for early-stage PCa and thus expand on the results from the previous study. Based on our results we speculate that at very low CNA burden, stochastic processes might influence the likelihood for either losses or gains to occur. However, as disease progresses and CNA burden increases towards the median, tumor cells with genomic losses might be preferentially selected as they might confer a competitive advantage. As tumor develops further, appreciable survival benefits might not be conferred by additional losses, whereas additional genomic gains might prove to be more advantageous. At very high CNA burden observed beyond the third quartile, stochastic processes might play a bigger role in determining occurrences of genomic gains or losses, resulting in a levelling-off of the number of losses to gains.

Combining the cost-effectiveness of LPWGS and the frequent availability of FFPE samples allows for the retrospective analysis of archival tumor tissue, which could benefit translational clinical research. Such an approach has already been shown to be useful in ovarian carcinoma^17^, where novel copy number signatures derived from shallow WGS (0.1 ×) were reported to predict OS and response to treatment. Our attempt to signature creation based on copy number calls from sequencing coverage as low as 0.02 ×, readily captured the most discriminatory copy number features (e.g. CN, SS). Two of these signatures (S1 & S2) were associated with worse BCR-free survival, and shed light into the complex architecture of copy number alterations. Notably, both the adverse signatures captured shorter segment sizes, and not surprisingly, higher number of breakpoints (either per 10 Mb or per chromosome arm). While it is fathomable that the major driver to clinical outcome in these signatures from our study were the copy numbers themselves, these results are encouraging for the use of this method for more routine CNA profiling.

Our results indicate this cost-effective methodology as a promising alternative to deep WGS for CNA profiling of FFPE prostate tumor tissue in the clinical setting. LPWGS at 0.1 × genome coverage allows for the analysis of 200 times more samples compared to WGS at an average coverage of 20 × and 20 times more samples compared to WES at an average coverage of 50 ×, at the same sequencing price, when using a Novaseq SP flowcell. Currently, this amounts to a per sample sequencing cost of roughly US$1150 for WGS, US$100 for WES and US$5 for LPWGS at the above-mentioned coverage. Additionally, costs for data storage, data backup and data analysis could be significantly lower for LPWGS compared to either deep WGS or deep WES. The exact price for these varies depending on the facility.

Our study has some limitations. First, we have assessed CNA from FFPE tumor tissue with the LPWGS using only one RP cohort. However, this was a comparatively large RP cohort with long clinical follow-up and complete clinical data, making it an ideal cohort for this analysis. Second, some of the samples in our cohort had low sequencing coverage. However, we did not observe any association between sequencing coverage and CNA burden. Third, CNAs were estimated using only one pipeline. But the results were consistent with known CNA profiles of early stage PCa. Fourth, assessment of the concordance between CNA calls from matched deep WGS, deep WES and LPWGS was not performed in this study and should be performed in future studies to establish accuracy of the LPWGS method in comparison to the current standards. Fifth, prospective data is required to assess the true clinical utility of this approach.

## Conclusions

In summary, using a cohort of 187 RP patients, we generated copy number profiles from FFPE tumor tissue samples using the cost-effective LPWGS approach. We show consistent association of copy number calls and CNA burden with adverse clinical and pathological parameters. Recurrent alterations in 8q, 13q, and 16q were readily identifiable and were shown to associate with a higher risk of BCR. The results support the use of LPWGS of FFPE tumor tissue in copy number profiling for early-stage localized PCa.

## Materials and methods

### Study cohort

Our cohort consisted of 187 men with histologically verified, clinically localized PCa. Patients underwent curatively-intended radical prostatectomy (RP) at the Department of Urology, Aarhus University Hospital, Denmark (1997–2009). Archived FFPE tumor specimens were obtained from Department of Pathology, Aarhus University Hospital. Written informed consent was obtained from all patients for the inclusion of prostate specimens in a research biobank (see section on ethical approval).

### DNA library preparation and LPWGS

For library preparation, DNA was extracted from FFPE prostate tumor tissue from punch biopsies having more than 80% cancer cells using the gDNA Eliminator columns from the RNeasy plus micro kit (Qiagen) as described before^21^. Libraries were prepared as described elsewhere^22^ using the Kapa Hyper Library Preparation Kit (KAPA Biosystems) and sequenced on an Illumina Novaseq instrument. We aimed for a coverage ≥ 0.05 × and a target coverage of 0.1 ×.

### Copy number estimation

Sequencing reads were aligned to the human reference genome hg38. Aligned bam files were imported to R and processed using CNAclinic^12^. An optimal bin size (50 kb) was selected based on the output from the *optimalBinsize* function maximising for the cross-validation log-likelihood and aiming for 30–180 reads per bin. Circular binary segmentation was performed using *runSegmentation*, with hg38 as the genome build. Gains were called if the log_2_ copy ratio for a segment was above a threshold of 0.15, and losses were called if the log_2_ copy ratio for a segment was below the threshold of -0.15. Genome–wide copy number profiles were plotted using *plotSampleData* and verified for known PCa-associated CNAs. Copy number calls were extracted using *exportData*. Median absolute pair-wise difference (MAPD) and CNA burden (fraction genome altered; FGA) were obtained using *statsCNA*. FGA was converted into PGA for further analyses.

### Statistical and data analysis

All statistical analyses were performed in R v4.0.3^23^ using RStudio build 386 (Posit software, PBC). Kruskal–Wallis, Wilcoxon, and log-rank tests were used to compare differences or associations between groups. BCR was used as endpoint for uni- and multivariate cox regression and Kaplan–Meier (KM) survival analyses. Boxplots, forest plot, and KM plots were generated using *ggboxplot*^24^, *ggforest*^25^, and *ggsurvplot*^25^, respectively. Basic plots were generated using *ggplot*^26^ or base R functions. Cohort-wide copy number frequency plot across each 50 kb bins was generated using *cnFreq*^27^. Copy number feature extraction and non-negative matrix factorization (NMF) was performed using the sigminer v2.0.4^11^ and NMF^28^ R packages. Eighty components across eight features were used for signature profiling. As described before^11^, these features are the number of breakpoints per 10 Mb (BP10MB) and per chromosome arm (BPArm), the absolute segmental copy number (CN), the copy number change point between adjacent segments (CNCP), the lengths of oscillating copy number segment chains (OsCN), the size of the copy number segment (SS) on a log_10_ scale, the minimal number of chromosomes with 50% copy number alterations (NC50), and the burden of copy number events in each chromosome (BoChr). Cophenetic and reconstruction (RSS) scores were used for assessing the optimal number of clusters, maximising for the cophenetic score and minimising the RSS error.

### TCGA-PRAD data analysis

The TCGA-PRAD SNP array copy number segment data was downloaded using the *TCGAbiolinks*^29^ R package for the primary tumor samples (n = 497). Copy number gains were called if the segment mean value was above 0.3 and the number of probes above 50. Similarly copy number losses were called if the segment mean value was below -0.3 and the number of probes were above 50. Genomic regions were annotated using the *biomart*^30^ and *GenomicRanges*^31^ R packages, with the type of overlap set to “within”. TCGA-PRAD WES copy number data was obtained from a previous study^11^ that re-analysed the WES data to call absolute copy numbers. In total, 498 TCGA-PRAD cases from primary tumors were included in this prior study^11^. We called copy number gains in this data if the absolute copy number was above 2 and called losses if the absolute copy number was below 2. Genomic regions were annotated as described above.

### Ethical approval and patient consent

All research forming the basis of this study was conducted in accordance to relevant rules, regulations, and guidelines and conform to the principles of the Declaration of Helsinki. Written informed consent was obtained from all participants prior to their donation of tissue samples for a research biobank, which was approved by The Central Denmark Region Committees on Health Research Ethics (jr. nr. 2000-0299) and The Danish Data Protection Agency (jr. nr. 2013-41-2041 and jr. nr. 2007-58-0010). The present study was approved by the Danish National Committee on Health Research Ethics (jr. nr. 1302791 and jr. nr. 1603542), who waived the requirement for patient consent to the specific analyses in this retrospective study.

### Supplementary Information

Supplementary Figures.

## Data Availability

Sequencing data generated in this study is available through controlled access from GenomeDK (https://genome.au.dk/library/) under accession number GDK000004 (https://genome.au.dk/library/GDK000004/).
