# Supplementary material for: Association between copy number alterations estimated using low-pass whole genome sequencing of formalin-fixed paraffin-embedded prostate tumor tissue and cancer-specific clinical parameters
Source: Sci Rep. 2023 Dec 17;13:22445. doi: 10.1038/s41598-023-49811-w (PMC10725894; doi:10.1038/s41598-023-49811-w)
Supplement: Supplementary file 1 — Supplementary Figures. [file 41598_2023_49811_MOESM1_ESM.docx]

**Supplementary Information for**

**Association between copy number alterations estimated using low-pass whole-genome sequencing of formalin-fixed paraffin-embedded prostate tumor tissue and cancer-specific clinical parameters**

**Paul Vinu Salachan^1,2^, Benedicte Parm Ulhøi^3^, Michael Borre^4^, Karina Dalsgaard Sørensen^1,2*^**

^1^Department of Molecular Medicine, Aarhus University Hospital, Aarhus N, Denmark

^2^Department of Clinical Medicine, Aarhus University, Aarhus N, Denmark

^3^Department of Pathology, Aarhus University Hospital, Aarhus N, Denmark

^4^Department of Urology, Aarhus University Hospital, Aarhus N, Denmark

*kdso@clin.au.dk (corresponding author)

**Supplementary Figures**


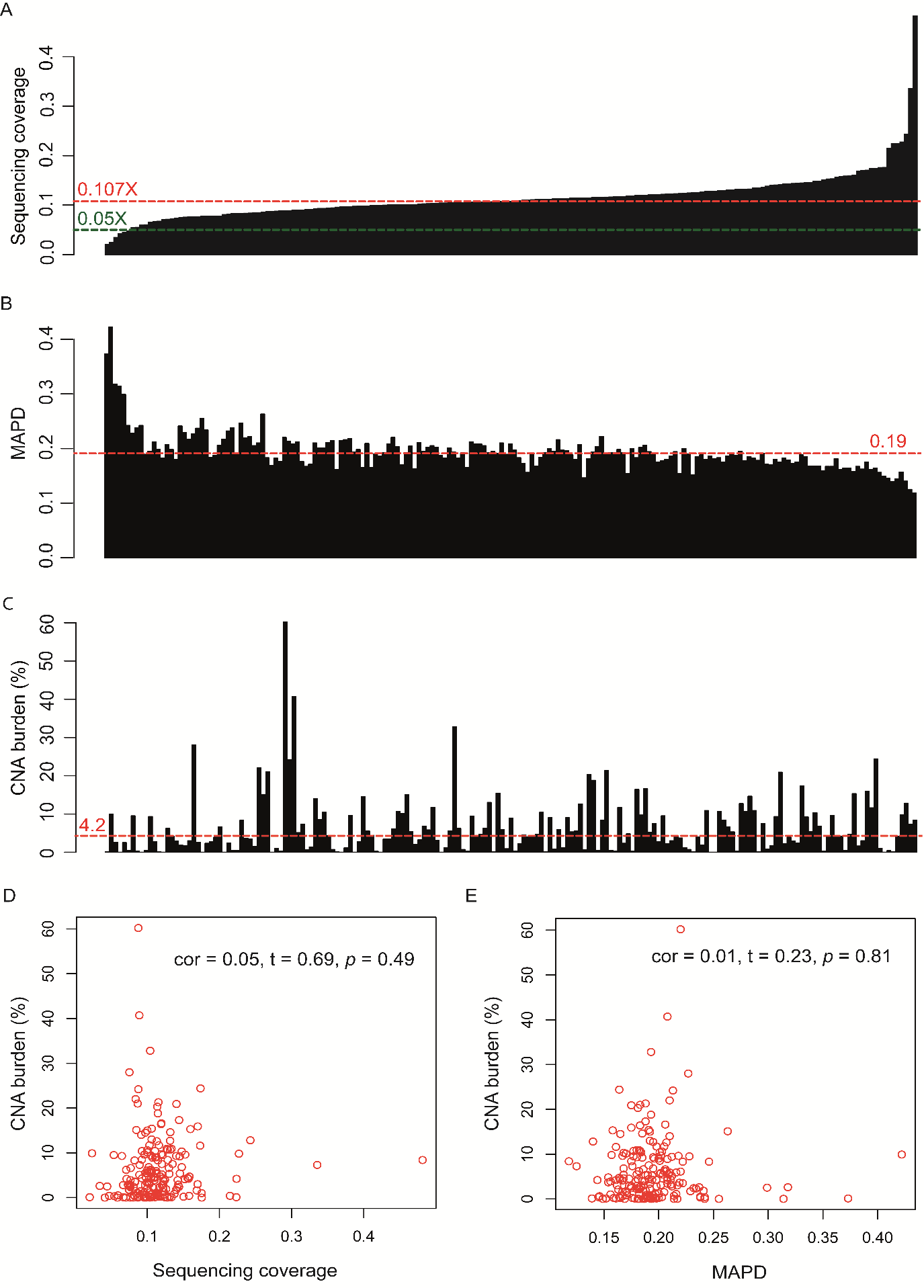


**Figure S1.** A) Sequencing coverage for each sample in the cohort. Red dashed line indicates the median coverage (0.107X). Green dashed line indicates the coverage aimed for in this study (0.05X). Except for six samples, all other samples had a coverage greater than 0.05X. B) Median absolute deviation pair-wise difference (MAPD) score for each sample in the cohort, arranged in the same order as in panel A. Red dashed line indicates median MAPD value (0.19). C) CNA burden (%) calculated for each sample in the cohort, arranged in the same order as in panel A. Red dashed line indicates median CNA burden (4.2 %). D) Correlation plot showing no correlation between CNA burden and sequencing coverage. E) Correlation plot showing no correlation between CNA burden and MAPD. Pearson’s product-moment correlation used for statistics.


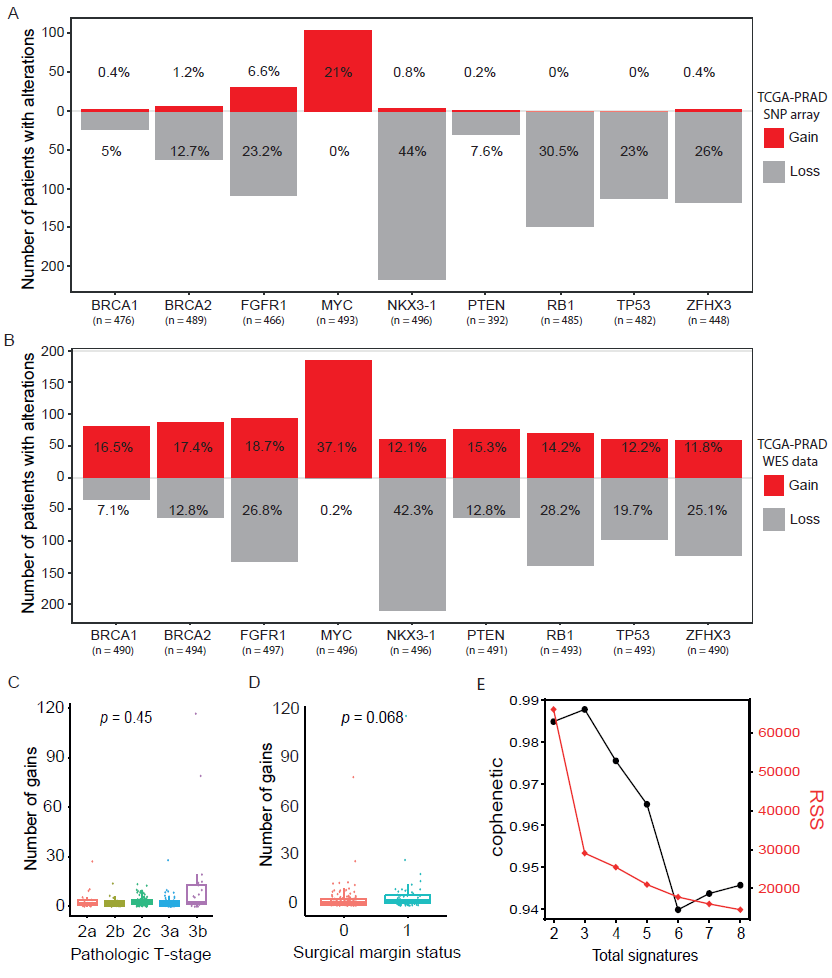


**Figure S2.** A). Number of patients in the TCGA-PRAD SNP array cohort (n=497) with alterations in genes that were previously reported to be recurrently affected by CNA in early stage PCa and located in regions 8p, 8q, 13q, and 16q. Copy number calls were inferred from SNP array data. B). Number of patients in the TCGA-PRAD WES cohort (n=498) with alterations in genes that were previously reported to be recurrently affected by CNA in early stage PCa and located in regions 8p, 8q, 13q, and 16q. Copy number calls were inferred from WES data. C) Boxplot showing number of gains per genome in different pathological T stages. *P*-value from Kruskal-Wallis test. D) Boxplot showing number of gains per genome in tumors with negative (0) vs. positive (1) surgical margin. *P*-value from Wilcoxon test. E) Cophenetic and RSS scores plotted for each signature number. Based on the high cophenetic and low RSS value, 3 clusters (signatures) were selected as optimum for signature extraction.
